# Sub-attosecond-precision optical-waveform stability measurements using electro-optic sampling

**DOI:** 10.1038/s41598-024-68848-z

**Published:** 2024-09-06

**Authors:** Syed A. Hussain, Christina Hofer, Maximilian Högner, Wolfgang Schweinberger, Theresa Buberl, Daniel Bausch, Marinus Huber, Ferenc Krausz, Ioachim Pupeza

**Affiliations:** 1 Max Planck Institute of Quantum Optics, Hans-Kopfermann-Str. 1, 85748 Garching, Germany; 2 Ludwig Maximilians University of Munich, Am Coulombwall 1, 85748 Garching, Germany; 3 Center for Molecular Fingerprinting, Molekuláris-Ujjlenyomat Kutató Közhasznú Nonprofit Kft., Budapest, Hungary; 4 Department of Physics and Applied Mathematics, Pakistan Institute of Engineering and Applied Sciences, Islamabad, Pakistan; 5 Department of Physics and Astronomy, University of British Columbia, Vancouver, BC Canada; 6 Quantum Matter Institute, University of British Columbia, Vancouver, BC Canada; 7 Physics Department and State Research Center OPTIMAS, University of Kaiserslautern-Landau, 67663 Kaiserslautern, Germany; 8 Department of Physics and Astronomy, King Saud University, 11451 Riyadh, Saudi Arabia; 9 Leibniz Institute of Photonic Technology Jena (IPHT Jena), Member of Leibniz Health Technologies, Jena, Germany

**Keywords:** Ultrafast lasers, Nonlinear optics, Infrared spectroscopy, Optical metrology

## Abstract

The generation of laser pulses with controlled optical waveforms, and their measurement, lie at the heart of both time-domain and frequency-domain precision metrology. Here, we obtain mid-infrared waves via intra-pulse difference-frequency generation (IPDFG) driven by 16-femtosecond near-infrared pulses, and characterise the jitter of sub-cycle fractions of these waves relative to the gate pulses using electro-optic sampling (EOS). We demonstrate sub-attosecond temporal jitter at individual zero-crossings and sub-0.1%-level relative amplitude fluctuations in the 10-kHz–0.625-MHz band. Chirping the nearly-octave-spanning mid-infrared pulses uncovers wavelength-dependent attosecond-scale waveform jitter. Our study validates EOS as a broadband (both in the radio-frequency and the optical domains), highly sensitive measurement technique for the jitter dynamics of optical waveforms. This sensitivity reveals outstanding stability of the waveforms obtained via IPDFG and EOS, directly benefiting precision measurements including linear and nonlinear (infrared) field-resolved spectroscopy. Furthermore, these results form the basis toward EOS-based active waveform stabilisation and sub-attosecond multi-oscillator synchronisation/delay tracking.

## Introduction

The ability to measure and control the temporal evolution of optical waves has spawned several fields of research and application over the past three decades. For instance, frequency-comb technology^1^ enables precision measurements of quantum transitions as well as the construction of high-precision optical clocks^2^. Attosecond metrology^3^ employs the electric-field oscillations of visible/near-infrared laser pulses to precisely clock photoelectron emission, permitting real-time access to electronic dynamics in matter.

Recently, the same ability has been leveraged in time-domain electric-field-resolved infrared metrology for unparalleled sensitivity and detection dynamic range in broadband molecular fingerprinting^4–6^. Here, parametric frequency down-conversion driven by state-of-the-art, high-power multi-MHz-repetition-rate, near-infrared (NIR) femtosecond lasers enables compact, temporally coherent radiation sources, covering large portions of the molecular fingerprint region with a brightness exceeding that of 3rd-generation synchrotrons^7–12^. Electro-optic-sampling^13–15^ (EOS) based field-sensitive detection—which has been routinely used for optical waves up to a few THz in laboratories and industry^16^—has recently been extended up to NIR frequencies^17^. In the mid-infrared (MIR), it has been shown to provide percent-level quantum efficiencies within the temporal gate^4^, with the prospect of approaching the fundamental single-photon detection limit^18^.

Recent works have demonstrated the potential of EOS-based field-resolved spectroscopy to overcome long-standing dynamic-range and sensitivity limitations of time-integrating spectroscopies (such as Fourier-transform spectroscopy) for molecular sensing in aqueous biological systems^4,6^ as well as gas-phase samples^5^. However, to further develop this spectroscopy toward its full potential, highly sensitive techniques for the measurement (and control) of the stability/reproducibility of the measured optical waveforms, together with a thorough understanding of the origins of (residual) waveform jitter, become necessary. Slow drifts of MIR waves have been studied with EOS previously, e.g. in ^19^. At shorter times scales, i.e. for single-shot characterization, methods like spectral interferometry and high-harmonic generation are limited by interferometer drifts and power fluctuations to 10 s or 100 s of mrad ^20,21^.

Here, we report the measurement of the fluctuations of sub-cycle fractions of a few-cycle MIR waveform spanning the 27–47-THz-band (at − 30 dB), time-gated by means of EOS, generated at a repetition frequency of 28 MHz and sampled at a rate of 1.25 MHz. For frequencies > 10 kHz, we measure relative amplitude and absolute zero-crossing timing jitters as low as 0.05% and 0.8 as (root-mean-square, RMS) respectively. By chirping the MIR pulse, we show that EOS permits measurements of attosecond-level wavelength-resolved waveform jitter, revealing dynamics of the parametric MIR generation process. These results showcase our EOS-based approach as a highly sensitive, broadband and wavelength-resolved optical-waveform-stability measurement technique. The outstanding waveform stability revealed by this method constitutes an essential milestone en route toward qualitatively new performance regimes of EOS-based field-resolved spectroscopy.

**Figure 1:**
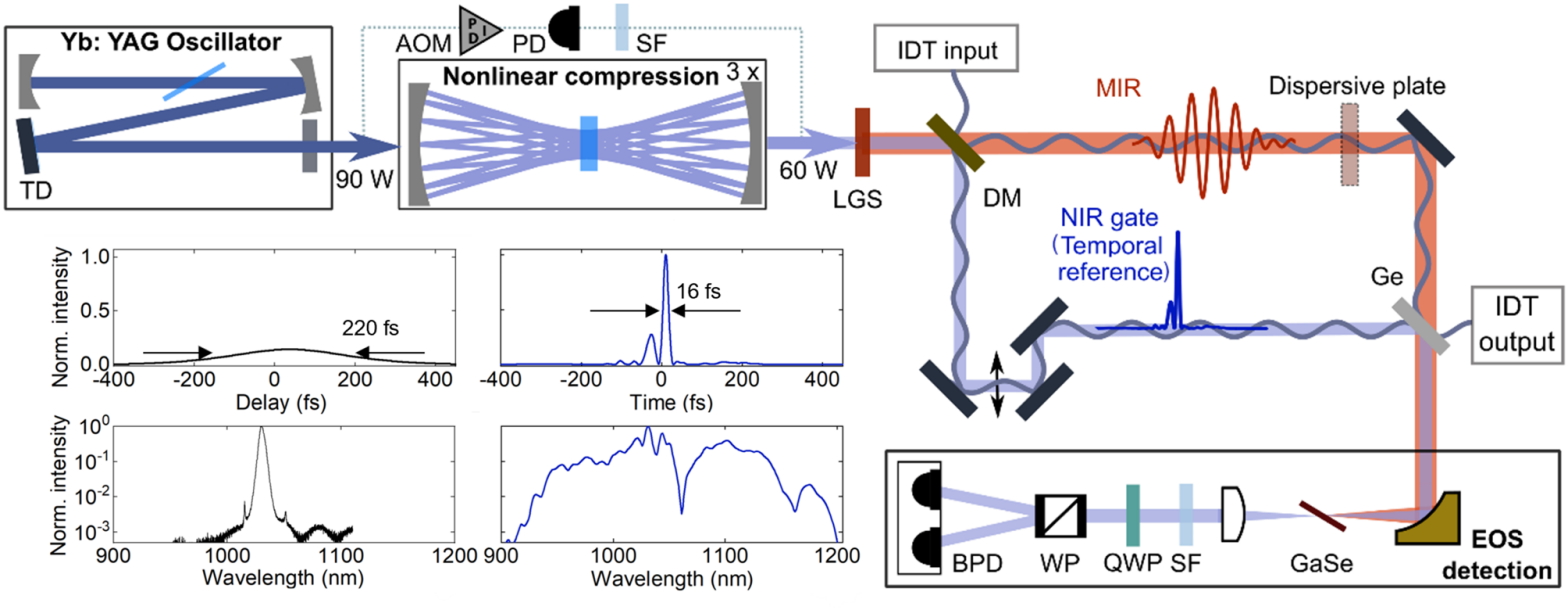
Schematic of the experimental setup. Initial pulses are provided by a Kerr-lens-mode-locked master oscillator, and temporally compressed in three stages, each consisting of a multi-pass self-phase-modulation cell followed by chirped mirrors. After nonlinear compression, the spectrally-filtered (SF) blue part of the near-infrared (NIR) is used to generate an error signal for intensity noise suppression with an acousto-optic-modulator (AOM)-based active stabilisation. The compressed NIR pulses drive IPDFG in a LiGaS_2_ (LGS) crystal. The NIR pulses are split from the emerging MIR beam with a dichroic mirror (DM), attenuated, temporally recompressed and used as gate pulses for EOS detection. Their envelope provides the temporal reference for the measurement. An auxiliary laser for interferometric delay tracking (IDT), coupled in at the DM, co-propagates along the NIR and MIR arms towards the EOS detection; t, time; I, intensity; λ, wavelength; PD, photodiode; Ge, germanium; GaSe, gallium selenide; SF, spectral filter; QWP, quarter-wave plate; WP, Wollaston prism; BPD, balanced photodiode.

In our experiment (Fig. 1 and Methods), the 220-fs NIR pulses emitted at 28 MHz by an Yb:YAG thin-disk oscillator are spectrally broadened via self-phase modulation in multiple passes through bulk fused silica and temporally compressed to 16 fs using chirped mirrors. They subsequently drive intra-pulse difference-frequency generation (IPDFG) in a LiGaS_2_ crystal. This results in close-to-octave-spanning MIR waveforms, spectrally centred at 8.1 µm, with an average power exceeding 60 mW. In a broadband phase-matching configuration, the off-centre spectral components of the 16-fs NIR pulses predominantly contribute to IPDFG. Because these components are affected most by oscillator intensity fluctuations, an error signal for active intensity-noise suppression with an acousto-optic-modulator^22^ at the oscillator output is generated from the blue wing of the broadened NIR spectrum.

**Figure 2:**
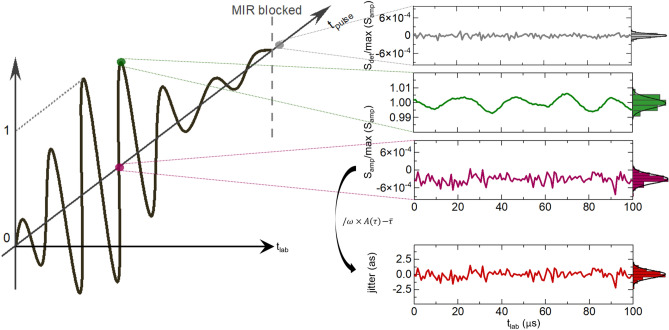
Measurement technique. Typical EOS trace in the MIR pulse time frame (${t}_{\text{pulse}}$). The EOS signal at fixed delays is measured for zero crossings (e.g., purple trace) and extrema (e.g., dark-green trace) in the laboratory time frame ${t}_{\text{lab}}$. For the blocked MIR beam, the detection noise is measured (grey trace). Panels on the right: EOS signals for: the detector noise, ${S}_{\text{det}}$, a (local) amplitude-maximum and zero-crossing, ${S}_{\text{amp}}$ (all normalised to the maximum amplitude ${\text{max}(S}_{\text{amp}})$) and the zero-crossing jitter (red trace), derived from the amplitude jitter via the slope and corrected with the IDT-measured position for the given zero crossing (see Methods).

Gate pulses for EOS are obtained from the NIR pulses transmitted through the IPDFG crystal, after temporal recompression. Their high average power (0.45 W), shot-noise-limited balancing and optimised spectral filtering after the EOS crystal^4,23^ afford low-noise, optical-field-sensitive detection. EOS traces of the MIR waveforms are recorded by varying the delay of the gate pulse with a mechanical stage (cf. axis ${t}_{\text{pulse}}$ in Fig. 2), permitting the determination of the delay values for individual signal extrema and zero-crossings. The geometric pathlength difference is monitored with the help of an auxiliary, frequency-stabilised continuous-wave laser^24^ enabling attosecond-precision delay tracking (Fig. 1 and Methods). As a measure for waveform stability, variations of the EOS signal at fixed delay-stage positions and therefore nominally constant gate-pulse delays are recorded at a sampling rate of 1.25 MHz (cf. axis ${t}_{\text{lab}}$ in Fig. 2), limiting the maximum measurable frequency to 0.625 MHz (Nyquist theorem). High-sensitivity EOS detection together with the MIR power level of several tens of mW results in a high detection dynamic range, which, in turn, affords waveform-stability measurements with high sensitivity.

**Figure 3:**
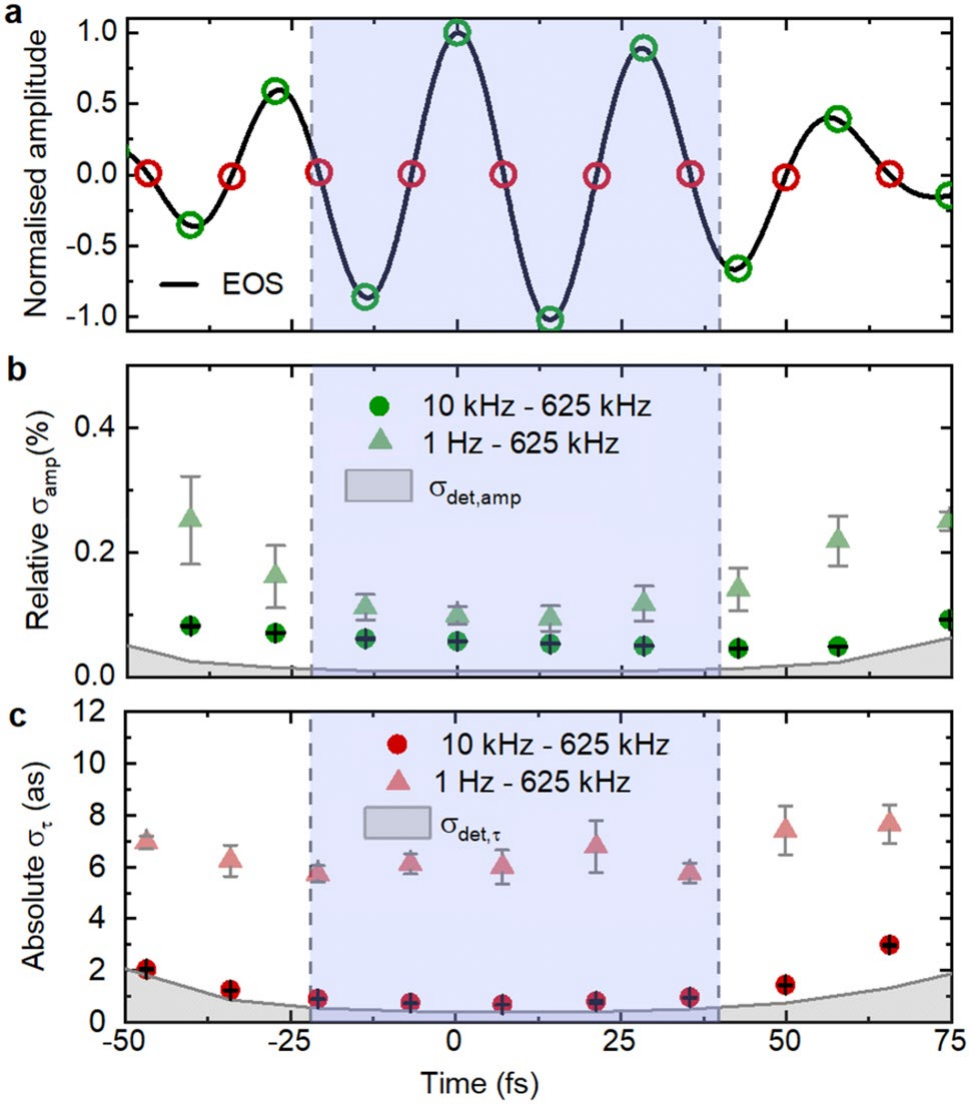
Measurement of waveform fluctuations for a compressed MIR pulse. (**a**) Measured few-cycle EOS trace. Empty circles: Selected waveform extrema (green) and zero crossings (red) for measurements in (**b**) and (**c**). Blue shaded area: Range of full-width-at-half-maximum intensity of the MIR pulse (62 fs). (**b**) Measured amplitude noise across the waveform for the 10-kHz-to-0.625-MHz band (green circles). Grey shaded area: detection noise level (i.e., sensitivity) for the measurement of relative amplitude fluctuations, determined by the dynamic range of EOS detection (see Methods). (**c**) Temporal jitter for each selected zero-crossing of the EOS trace, in the 10-kHz-to-0.625-MHz band (red circles). Grey shaded area: Sensitivity for timing jitter measurements (see Methods). Error bars in (**b**) and (**c**) indicate the standard deviation of five measurements each.

Figure 3b,c shows statistics for the relative amplitude jitter ${\sigma }_{\text{amp}}$ and absolute zero-crossing timing jitter ${\sigma }_{\tau }$ (Fig. 1, Methods) of the temporally-compressed waveform (Fig. 3a), obtained from repeated measurements at selected delay values. For the cycles within the intensity full width at half maximum (FWHM), we obtain mean values of ${\sigma }_{\text{amp}}\hspace{0.17em}=\hspace{0.17em}0.05\text{\%}$ and ${\sigma }_{\tau }=0.8$ as, respectively, in the 10 kHz–0.625 MHz frequency band. These are, on average, 5 and 1.8 times above the detector sensitivity for the intensity and absolute zero-crossing timing jitter, ${\sigma }_{\text{det},\text{amp}}$ and ${\sigma }_{\text{det},\tau }$ (grey shaded areas in Fig. 3b,c), respectively. It is noteworthy that a temporal jitter of 0.8 as corresponds to a phase jitter of 0.18 mrad at the centre wavelength of the pulse of 8.1 µm. For comparison, the lowest RMS values measured for the carrier-envelope-phase jitter of ultrashort-pulse trains are 15 mrad^25^ (in the 1 Hz–2.5 MHz-band) and 20 mrad^26^ (bandwidth not reported) for active and passive phase stabilisation, respectively.

Including the frequencies between 1 Hz and 10 kHz increases the jitter values (< 0.3%, < 10 as, triangles in Fig. 3), presumably due to the influence of mechanical/acoustic noise on the entire setup. The jitter values measured on these time scales are commensurate with those reported in Fig. 4 of Ref.^4^. However, intensity and group-delay jitter in this frequency region can be reduced in postprocessing by scanning faster than the noise^27–29^.

**Figure 4:**
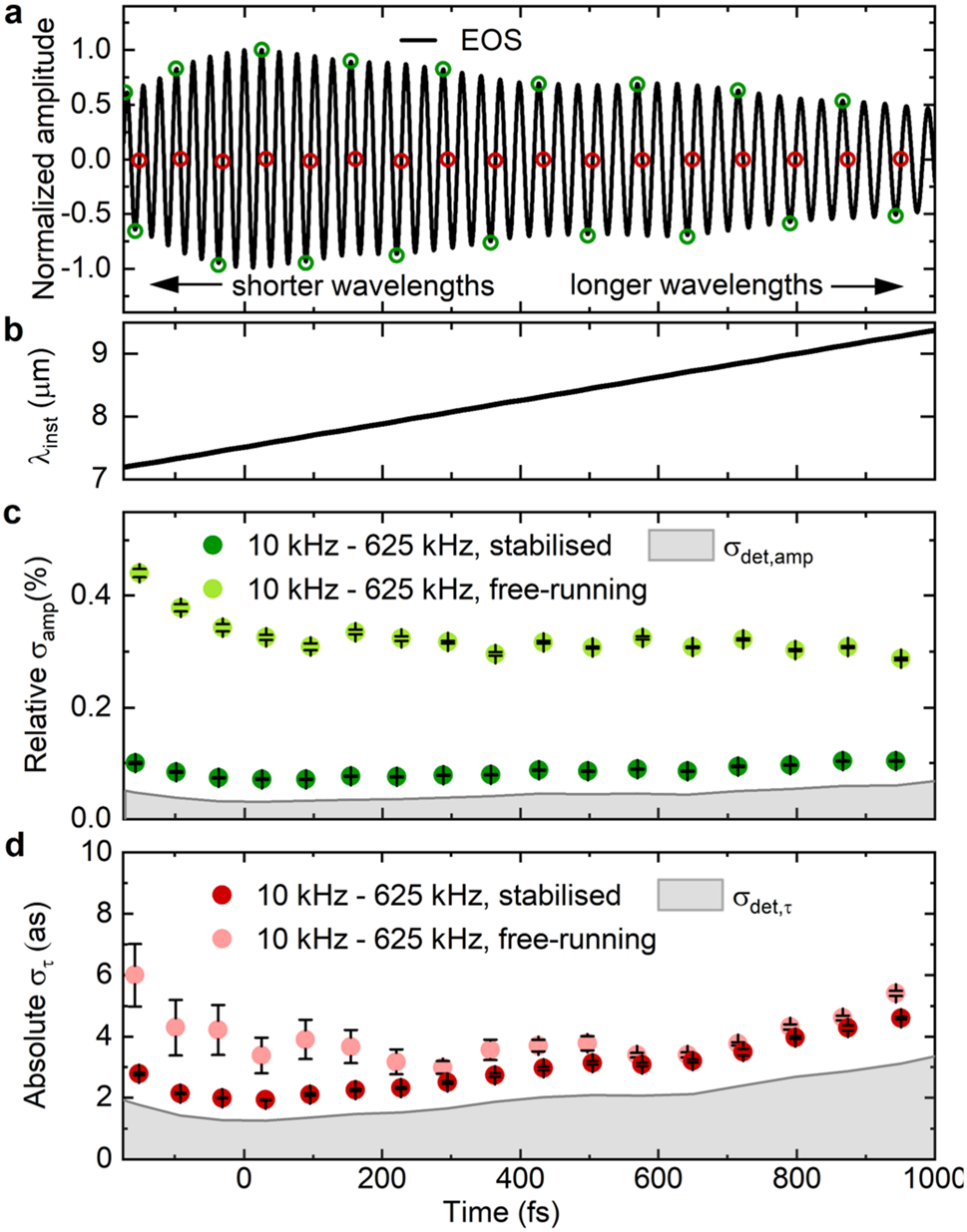
Measurement of waveform fluctuations for a chirped MIR pulse. (**a**) Measured EOS trace of chirped pulse. Empty circles: selected zero-crossings (red) and extrema (green) for the measurements in (**b**) and (**c**). (**b**) Instantaneous wavelength over the chirped pulse. (**c**) Measured amplitude noise across the pulse, for the 10-kHz-to-0.625-MHz band, with the AOM-stabilisation on (dark green circles) and off (light green circles). (**d**) Temporal jitter for each selected zero-crossing of the MIR waveform, in the 10-kHz-to-0.625-MHz band, with the AOM-stabilisation on (dark red circles) and off (light red circles). Grey shaded areas: detection limit, analogous to Fig. 3. Error bars in (**c**) and (**d**) indicate the standard deviation of five measurements each.

To demonstrate the ability of our method to sensitively characterise waveform stability in a wavelength-resolved manner, we have inserted a 5 mm-thick CaF_2_ plate in the MIR beam path, dispersing the wavelengths along the (increased) duration of the pulse (Fig. 4a,b). For both amplitude (Fig. 4c, dark green) and zero-crossing fluctuations (Fig. 4d, dark red), we observe a steady decrease with decreasing wavelength. We attribute this behaviour to the active NIR intensity stabilisation^22^, whose error signal is derived from the blue wing of the nonlinearly-broadened spectrum, which in IPDFG contributes more to the generation of shorter MIR wavelengths. This is confirmed by the result of turning off the active intensity stabilisation, which increases both the amplitude and zero-crossing fluctuations predominantly for the shorter MIR wavelengths (Fig. 4c,d, light green/red). It is noteworthy that variations in the mean values of the zero-crossing jitter of less than 1 as can be measured in a wavelength-resolved fashion.

The significance of our study is twofold. Firstly, it validates EOS as a high-sensitivity and broadband measurement technique for optical-waveform stability. In the optical domain, using GaSe as a nonlinear material with broadband phasematching allows for measurements over a wide spectral range. In the radio-frequency domain, measurements at fixed delays with a sampling rate of 1.25 MHz enable the characterization of the fluctuations at various timescales. The presented method has distinct differences to traditional optical-phase-noise measurement techniques^30,31^. Most importantly, here (1) the timing reference is determined by the gate pulse envelope, and (2) sub-optical-cycle nonlinear gating grants access to wavelength-dependent temporal jitter within broadband waveforms. In addition, the EOS-based method employs lowest-order nonlinearities to generate a signal that is linearly proportional to the MIR electric-field strength and to the gate pulse intensity. Harnessing the ~ 10-fs-scale pulses of modern, high-power NIR femtosecond lasers for gating and using a spectral filter after the EOS crystal^23^ results in an exceptional sensitivity, without the need for high MIR field strengths. In the future, EOS could also be used to actively stabilise optical waveforms and to facilitate low-noise coherent waveform synthesis^32^.

Secondly, the stability level reported here for waveforms generated via IPDFG unveils avenues toward qualitatively new performance regimes for field-resolved infrared metrology. For instance, assuming the acquisition of multi-picosecond EOS traces at rates > 10 kHz^27–29,33^ and statistical averaging of electric-field molecular fingerprints^29^ over measurement times on the order of several seconds promises to reduce the amplitude and zero-crossing jitter by roughly 3 orders of magnitude with respect to state-of-the-art condensed-matter measurements^4^. In particular, fast acquisition via electronically-controlled dual-oscillator optical sampling schemes can benefit from the outstanding waveform stability demonstrated here, by using EOS for temporal referencing with sub-attosecond precision^27^. This method can provide an orders-of-magnitude increase in timing accuracy compared to the state of the art^4,20,21,34^, translating to a corresponding improvement in sensitivity and/or precision for linear and nonlinear infrared spectroscopy.

## Methods

### Laser system and electro-optic sampling

The laser system is described in detail in Ref.^4^. In short, an Yb:YAG thin-disk, Kerr-lens-modelocked oscillator delivers a 28 MHz-repetition-rate train of 220 fs full-width-at-half-intensity-maximum (FWHM) duration soliton pulses with 90 W of average power. Temporal compression via self-phase modulation distributed over several focused interactions with bulk fused silica (Herriott-cell-arrangement), and reflections off chirped multilayer mirrors results in a 60 W-average-power train of 16 fs-FWHM near-infrared (NIR) pulses, spectrally centred at 1.03 µm ^36^ (temporal intensity envelope from frequency-resolved optical gating measurement, shown in Fig. 1). A small portion of this broadened spectrum is short-pass-wavelength-filtered at 925 nm and used to derive an error signal for active intensity stabilisation with an acousto-optic modulator at the oscillator output^22^. The 60 W-pulses are focused onto a 1-mm-thick LiGaS_2_ crystal with a peak intensity of 180 GW/cm^2^, where they drive the second-order parametric nonlinear process of intrapulse difference-frequency generation (IPDFG) in the type-I phase matching configuration, generating close-to-octave-spanning MIR waveforms spectrally centred at 8.1 µm (37.01 THz) with an average power in excess of 60 mW^4^. The NIR and MIR pulses are separated by a dichroic beam splitter, after which 0.45 W of the NIR pulses are re-compressed to 16 fs to serve as gate pulses in EOS. The delay between the MIR and the NIR gate pulses is varied with a mechanical stage. A 3-mm-thick germanium plate acts as a beam combiner for the NIR and MIR pulses. An average MIR power of about 20 mW arrives at the EOS crystal. The NIR and MIR pulses are collinearly focused into an 85 µm-thick GaSe crystal, phase-matched for sum-frequency generation^15,35^. The resulting sum-frequency radiation is detected in a heterodyne fashion with a balanced detector, using a short-pass-filter at 912 nm to increase the signal-to-noise-ratio of the EOS detection^23^.

### Interferometric delay tracking (IDT) and data acquisition

To minimise the influence of interferometer jitter on the measurements of temporal waveform jitter, we track the delay in the two interferometer arms with an auxiliary laser, as described in detail in Ref.^21^. The beam of a frequency-controlled distributed-feedback laser diode emitting a continuous wave at 1550 nm is coupled into the interferometer arms at the dichroic mirror splitting the NIR from the MIR beam after IPDFG. It propagates along the interferometer arms with the exception of a 10 cm distance necessary to circumvent the germanium beam combiner that is opaque at 1550 nm. In contrast to a simple interference signal, frequency modulation allows for the determination of the delay direction^21^.

The EOS data (output of the balanced photodetector) are acquired with the IDT electronics and, thus, with a common clock, such that the EOS and position data are synchronised. A mechanical delay stage temporally scans the gate pulse across the entire MIR waveform. Once the positions of the waveform zero-crossings and extrema are determined, the EOS signal at a given delay position is recorded for one second with a sampling rate of 1.25 MHz. The slope of the EOS waveform at a given delay position is used to correct for the geometric path length jitter. For frequencies beyond 3 kHz, IDT position correction does not improve the zero-crossing statistics, such that the raw data (i.e., assuming a constant delay in the interferometer) are used for analysis above this frequency. The measurements are done sequentially for the different delay values and repeated five times for each point yielding the statistics indicated in Figs. 3,4.

### Sensitivity of the waveform stability measurements

The detection sensitivity was determined as follows: We measured the average amplitude value of the signal at the maximum of the EOS trace (i.e. at delay $\tau =0$), ${\overline{S}}_{\text{max}}\left(0\right)=4.8$ V and the standard deviation of the detection noise (3 × 10^–4^ V, with blocked MIR beam). With both signals normalised to ${\overline{S}}_{\text{max}}\left(0\right)$, the noise corresponds to the minimum detectable relative amplitude change ${\sigma }_{\text{det},\text{amp}}\left(0\right)=6\times {10}^{-5}$. For delay values different from $\tau =0$, this value is increased according to the normalised electric field envelope amplitude $\widehat{A}\left(\tau \right)$: ${\sigma }_{\text{det},\text{amp}}\left(\tau \right)={\sigma }_{\text{det},\text{amp}}\left(0\right)/\widehat{A}\left(\tau \right)$.

We measured the timing jitter by mapping the IDT-position-corrected amplitude jitter at a zero-crossing position to the time domain via the average slope of the optical waveform with angular frequency $\omega$ and amplitude *A* (approximation: $A\times sin\left(\omega \tau \right)\approx A\omega \tau$ around the zero-crossing): $\Delta S\left(\tau \right)= A\left(\tau \right)\times \omega \times \Delta \tau$. Therefore, the minimum detectable timing- and amplitude-jitter values are related as follows: ${\sigma }_{\text{det},\uptau }\left(\tau \right)=\frac{{\sigma }_{\text{det},\text{amp}}\left(\tau \right)}{\omega }$, resulting in ${\sigma }_{\text{det},\tau }\left(0\right)\approx 0.5$ as.

## Data Availability

The data underlying the figures in this paper are available from the corresponding author upon reasonable request.
